# Feasibility of autologous plasma gel for tonsil-derived stem cell therapeutics in hypoparathyroidism

**DOI:** 10.1038/s41598-018-30454-1

**Published:** 2018-08-09

**Authors:** Soo Yeon Jung, Ha Yeong Kim, Hyun Ju Oh, Euno Choi, Min Sun Cho, Han Su Kim

**Affiliations:** 1 Department of Otorhinolaryngology - Head and Neck Surgery, College of Medicine, Ewha Womans University, Seoul, Republic of Korea; 2 Department of Molecular Medicine, College of Medicine, Ewha Womans University, Seoul, Republic of Korea; 3 Department of Pathology, College of Medicine, Ewha Womans University, Seoul, Republic of Korea

## Abstract

Hypoparathyroidism is a deficiency of the parathyroid hormone (PTH) in the body. We previously reported the possibility of treating it using tonsil-derived mesenchymal stem cells (TMSCs) differentiated into PTH-releasing cells. The purpose of this study was to evaluate the feasibility of using autologous plasma gel as scaffold material in treatment of hypoparathyroidism with TMSC. We obtained plasma by venous sampling of autologous blood and centrifuged and fabricated the plasma gel using a sinusoidal pattern heating machine. After we created the hypoparathyroidism animal model, we administered undifferentiated TMSCs and TMSCs differentiated into parathyroid cells at each rat dorsum by intramuscular injection with and without the plasma gel. In the plasma gel groups, intact PTH was detected from on day 21 after TMSC injection; we did not detect intact PTH in the groups that were only transplanted with TMSCs during the entire experimental period. Serum calcium was higher and phosphorous was lower in the TMSC with plasma gel groups than in the groups with TMSCs alone. We detected PTH and chromogranin A in the TMSC-plasma gel-transplanted areas on immunohistochemistry and immunofluorescence stain. Plasma gel can be considered as a cell-delivery scaffold for treating hypoparathyroidism with tonsil-derived mesenchymal stem cells.

## Introduction

Hypoparathyroidism is an endocrine deficiency that originates from low parathyroid hormone (PTH) levels. The conventional treatment has been a 1,25-dihydroxyvitamin D calcium supplement, but this therapy causes discomfort to patients because of the side effects from the large amount of additional calcium intake. Furthermore, calcium and vitamin D cannot perfectly replace PTH in balancing human mineral levels, and long-term bone morphologic changes and nephrolithiasis could be induced^1,2^. The US Food and Drug Administration (FDA) recently approved injectable synthetic PTH (Natpara®, Shire-NPS Pharmaceuticals, Inc., Lexington, MA, USA) for treating osteoporosis and hypoparathyroidism^3,4^. This modality is physiologically ideal for delivering normal PTH, however, it has a short duration in the body and requires daily injections and it is expensive.

Transplantation of long-lasting, biocompatible hormone-releasing tissue in the body can be the ideal hormone replacement therapy (HRT) for hypoparathyroidism. Autologous parathyroid gland implantation could be ideal in cases of accidental parathyroid gland excision that is noticed during surgery^5,6^; however, when the unexpected removal of the gland is detected after surgery by histological evaluation, auto-transplantation could not be an option. To overcome this limitation, it is necessary to develop tissue-engineered PTH that can be easily manufactured and transplanted.

Hormone-secreting cells, growth factors, and extracellular matrix (ECM)-rich scaffolds that help cell survival and engraftment determine the quality of cell therapy. We previously reported on tonsil-derived mesenchymal stem cells (TMSCs) as a source of PTH-releasing cells and successfully differentiated them into PTH-releasing cells^7^. PTH-releasing cells derived from TMSCs have demonstrated promise in both *in vitro* and *in vivo* studies; unlike with conventional HRT, these cells have been found to regulate PTH secretion by automatically reacting to calcium concentrations. However, developing suitable cell-delivery materials has been difficult. In previous studies, we have applied small intestine submucosa substances(SIS)^5^, thermosensitive gels^8^, and Matrigel® (MA, BD Biosciences, San Jose, CA)^7^ as scaffolds for PTH-releasing cells; however, these biomaterials had some limitations. SIS had sol formulation which is easy for mixing with cells and injecting into body, however, SIS dissipated easily in the body. Human application is limited since SIS is xenogenic material. Thermosensitive gel has excellent physical features, but it is composed of complete artificial material (polyethyleneglycol-polyalanine-co-phenylalanine) that lacks growth factors and extracellular matrix (ECM) which both help cell engraftment. Matrigel® has proper formula as cell-carriers and abundant biofactors. These properties facilitate cell injection and engraftment. However, this material is not approved in human applications because it is gelatinous protein mixture secreted by Engelbreth-Holm-Swarm (EHS) mouse sarcoma cells.

Plasma gel (PG) is a biologic gel derived from blood; the plasma is easily obtained by blood sampling and centrifuge. PG is autologous material with notable advantages in clinical applications over other scaffolds; less risks of infection or rejection reaction and no necessary of separate FDA approval. In terms of fabrication methods, studies have been published on mixing PG with CaCl_2_, fibrin, other gels including hydrogel, and hyaluronic acid as well as heating it^9–13^; among these methods, heating has the advantages of shorter production time and lower cost. For cosmetic augmentation and vocal fold paralysis treatment, plasma gel fabricated by heating has been used, and its safety was demonstrated^12,14^. Plasma was also reported to provide excellent conditions for cell culture and differentiation because of the abundance of growth factors and ECM^10,15^.

The aim of this study was to assess the feasibility of plasma gel as an injectable TMSC-delivery scaffold for treating hypoparathyroidism by *in vitro* evaluation and *in vivo* animal experiment.

## Results

### Mechanical and physical properties of the plasma gel

**Figure 1:**
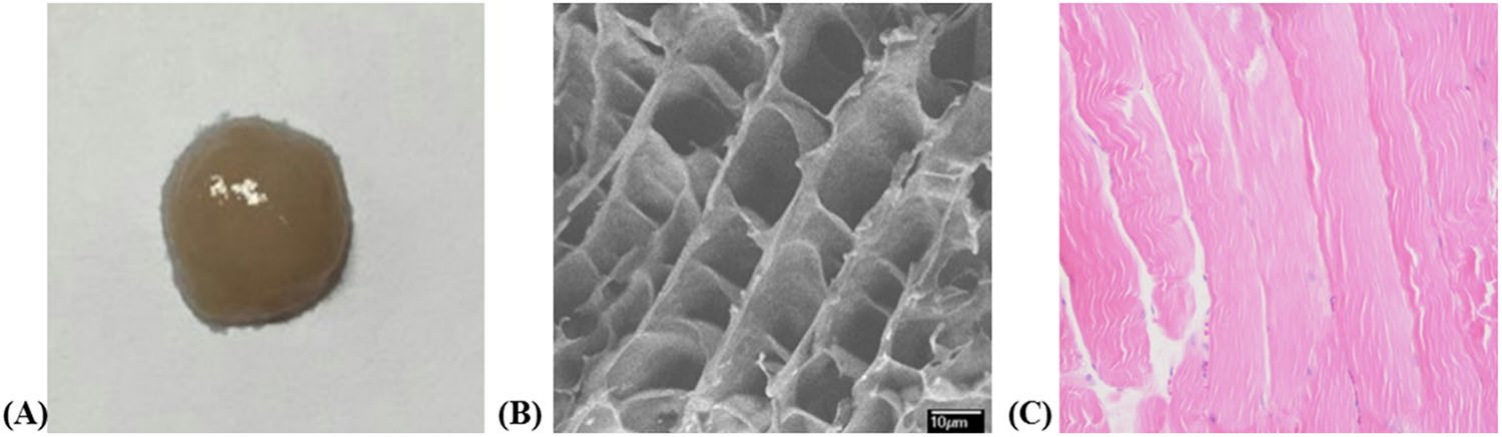
Morphological evaluation of fabricated plasma gel. (**A**) Fabricated plasma gel has translucent feature as gel. (**B**) Honeycomb shaped with 10 nm diameter pores were observed on scanning electron microscopic evaluation. (**C**) Plasma gel showed revealed regular protein material without nuclei on histology (H&E, ×200).

We successfully fabricated the plasma gels using the ALSA S-1; the fresh fabricated gels were whitish and translucent (Fig. 1A). We used the SEM images to observe the gels’ microstructure, and the PGs showed a multi-porous interconnected honeycomb structure with 10 nm × 10 nm^2^-sized pores (Fig. 1B).

**Figure 2:**
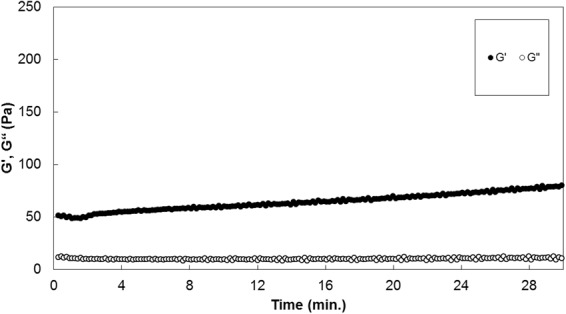
Rhelogical evaluation of the plasma gel. Storage modulus (G′) and loss modulus (G″) were 64.29 and 10.00 Pa at 37 °C (body temperature).

Rheological evaluation found that the mean storage modulus (G′) and loss modulus (G″) were, respectively, 64.29 and 10.00 Pa at 37 °C (body temperature). The G′ increased from 51.14 to 78.39 Pa during the experiment (30 min; Fig. 2).

### Intact PTH and serum calcium and phosphorus levels of the animals

**Figure 3:**
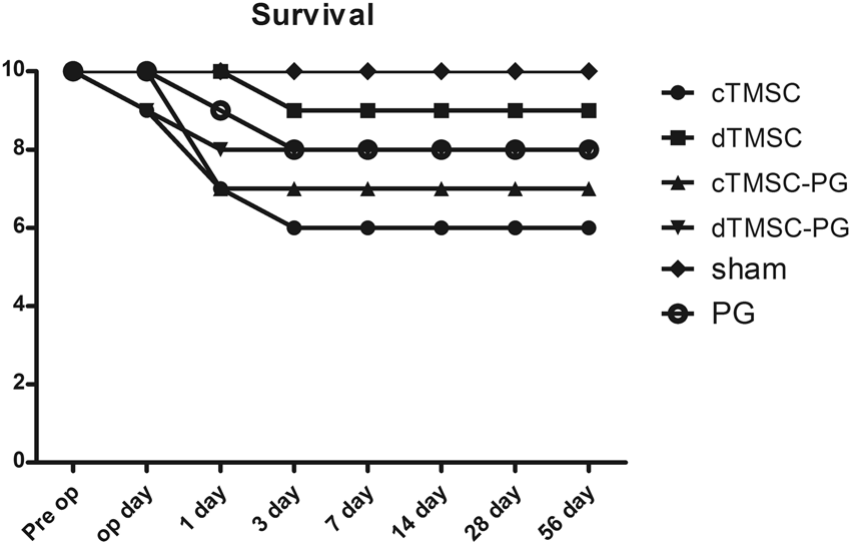
Survival of the transplanted animals. Animals expired before 3 postoperative days. There were no statistically significant differences between the groups.

Among the 60 animals, 9 died from surgery- or anesthesia-related problems within 24 hours of surgery. Three animals (each in cTMSC, dTMSC, and PG group) died within 24 to 48 hours postoperatively, and we detected no tetany or muscle spasm to reflect hypocalcemia at death. Survival rates did not show statistically significant differences among the groups (Fig. 3).

**Figure 4:**
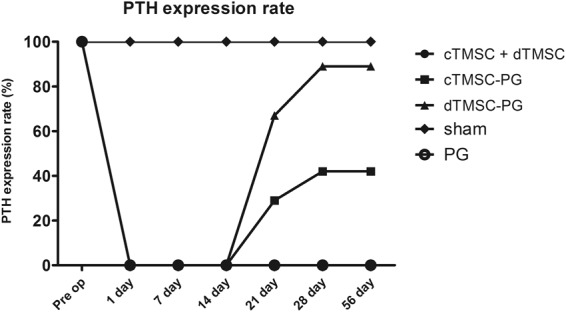
Intact parathyroid hormone in the groups. Intact parathyroid hormone (PTH) was not detected in all groups until postoperative 14 days. Twenty-one days after the transplantation, animals in the cTMSC-PG, dTMSC-PG, sham groups showed intact PTH, but the cTMSC, dTMSC, and PG only groups showed no detectable PTH during the experimental period.

Intact PTH decreased after parathyroidectomy from mean preoperative levels of 150.74 ± 127.76 pg/mL (ranges: 20.40–488.89) to being non-detectable at postoperative day 1 in all groups except the sham group. The sham group demonstrated intact PTH within normal rage during the whole experimental period. We detected intact PTH 21 days after the transplantations in the cTMSC-PG and dTMSC-PG groups, but in contrast, intact PTH remained non-detectable during the whole experimental period in the cTSMC, dTMSC and PG groups (Fig. 4). These results indicated that intact PTH levels were restored only in the groups for which we used the plasma gel as the cell-delivery scaffold. Intact PTH was restored to normal ranges in three animals (all in the dTMSC-PG group), whereas other animals showed increased intact PTH but not full restoration to normal levels. We observed increased intact PTH in seven of the eight animals that were alive after the surgery. In the cTMSC-PG group, we detected postoperative intact PTH in only three of the seven surviving animals. These results demonstrated that parathyroid-differentiated TMSCs were more effective for releasing the PTH *in vivo*. In our comparisons of the iPTH values, the dTMSC-PG group showed a higher mean than the cTMSC-PG group (Table 1), although the result was not statistically significant.

**Table 1:** Intact parathyroid hormone levels of the animals.

Intact PTH (pg/mL)	21 days	28 days	42 days	56 days	84 days
cTMSC-PG	2.40 ± 3.95	5.29 ± 3.83	9.15 ± 8.16	27.29 ± 17.47	14.67
dTMSC-PG	11.53 ± 11.38	24.12 ± 2.73	25.91 ± 10.04	21.29 ± 11.72	45.73 ± 27.40
Sham	147.22 ± 62.78	162.32 ± 32.85	103.42 ± 52.96	75.57 ± 42.93	123.25 ± 89.21

After parathyroidectomy, serum calcium level decreased and phosphorus level increased (Table 2); calcium and phosphorus were not fully restored to preoperative levels in any of the groups. When we compared the effects of the PG on TMSC transplantation, calcium was higher and phosphorus was lower in the groups that received the TMSCs transplanted with the plasma gel (cTMSC-PG and dTMSC-PG) than in the groups that were transplanted with TMSCs only (cTMSC and dTMSC), and the differences between the two groups (with and without PG) were statistically significant. Comparing the effects of TMSC type on calcium and phosphorous levels found that differentiated TMSCs showed higher mean calcium and lower mean phosphorous than did the undifferentiated TMSCs, but there was no statistically significant difference (Table 2).

**Table 2:** Serum calcium and phosphorus levels of the animals.

	Serum calcium (mg/dL)	Serum phosphorus (mg/dL)
Pre operation		
all groups	10.50 ± 0.53	7.47 ± 0.67
Postoperative 8 weeks		
Sham	9.82 ± 0.30	7.64 ± 0.38
PG	5.74 ± 0.80	14.46 ± 0.71
cTMSC	5.51 ± 0.46	13.99 ± 0.86
dTMSC	6.19 ± 0.87	12.83 ± 2.62
cTMSC + dTMSC	5.85 ± 0.74	13.41 ± 1.91
cTMSC-PG	6.34 ± 0.90	13.29 ± 0.82
dTMSC-PG	7.35 ± 0.77	9.29 ± 1.77
cTMSC-PG + dTMSC-PG	6.92 ± 0.93	10.63 ± 2.51
**P-value**	0.040*	0.043*

Data are expressed as the mean ± standard deviation.

*p-value < 0.05 in Mann-Whitney test between the cell-only implanted groups (cTMSC & dTMSC) and cell implanted with plasma gel groups (cTMSC-PG & dTMSC-PG) postoperative 8 weeks results.

### Assessment on implanted TMSC with PG

**Figure 5:**
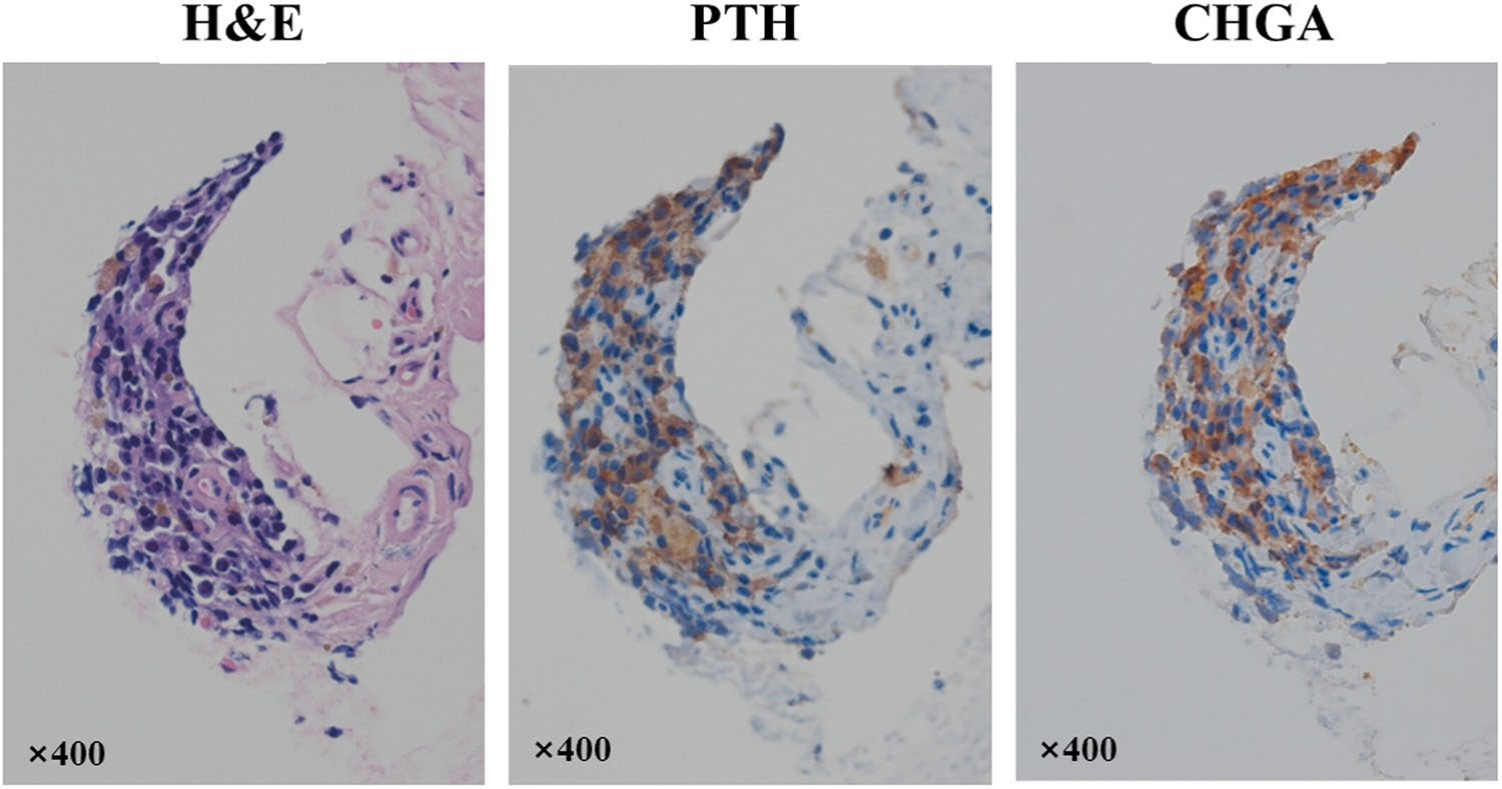
Histological evaluation of transplanted tonsil-derived stem cells with plasma gel. The tissue in the transplantation sites is composed of the cells having round nuclei with scant to moderate amount of eosinophilic cytoplasm (H&E, x400). Immunohistochemical staining for parathyroid hormone (PTH) and chromogranin A (CHGA) showed the existence of the parathyroid tissue (×400).

**Figure 6:**
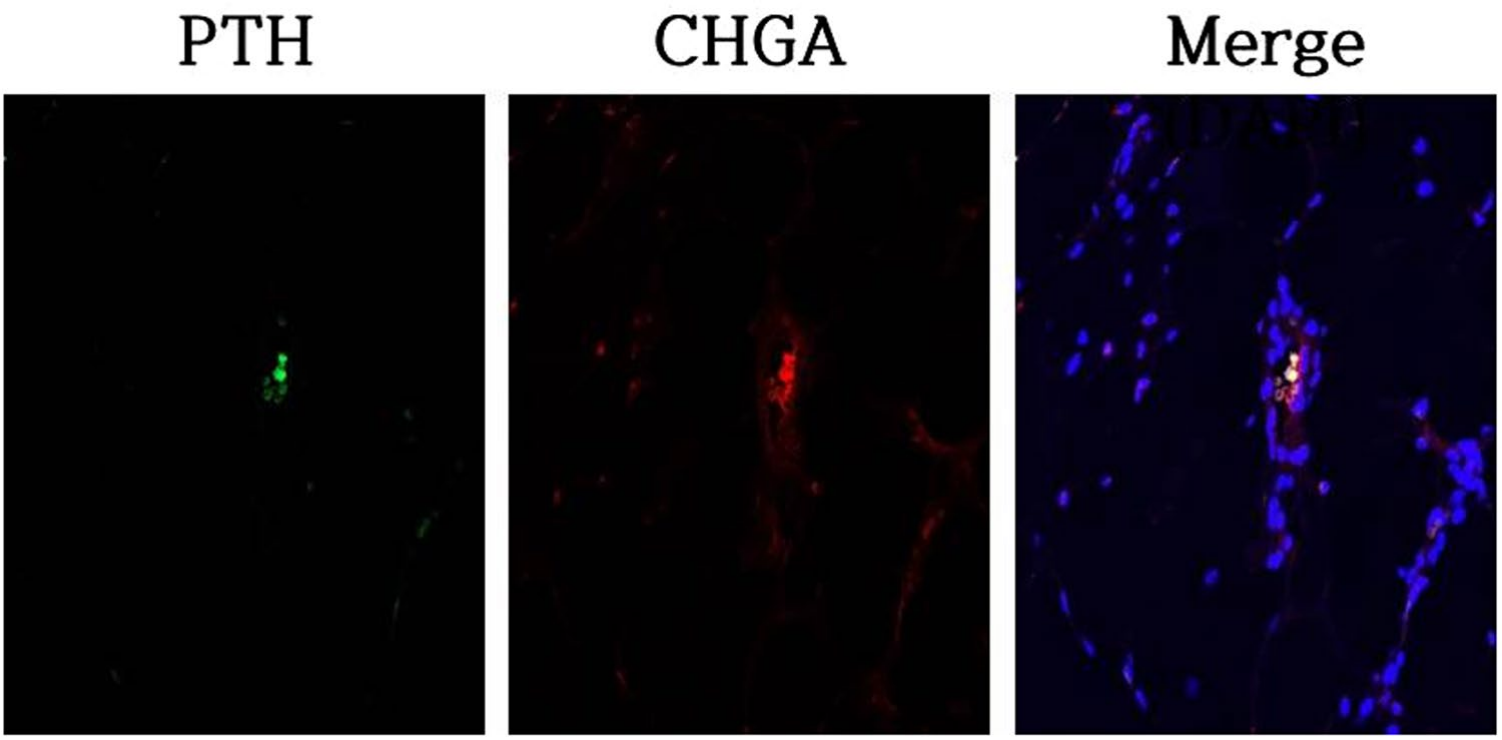
Immunofluorescence images of transplanted tonsil-derived stem cell with plasma gel. Parathyroid hormone (PTH) and chromogranin A (CHGA) staining revealed that parathyroid-secreting cells were alive in transplanted area on 56 days after the transplantation.

We noted the transplantation sites on the animals as the yellow-brownish tissue. We confirmed the parathyroid tissue on the immunohistochemical studies; however, it was difficult to identify the chief cell or the oxyphil cell which formed the normal parathyroid gland tissue in the H&E slides (Fig. 5). Immunofluorescent staining confirmed the presence of cells that expressed PTH and Chromogranin A (CHGA, a secretory granule marker protein) at the transplantation sites (Fig. 6).

## Discussion

Proper scaffold material is essential for maximizing the effects of newly developed cell therapy; ideal material should facilitate cellular engraftment and long-term hormone release. In order to be widely used, a scaffold should be easy to fabricate and inject, and it should be advantageous in terms of cost and absence of foreign matter reactions. In addition, it is advisable that scaffolds keep the cells in the injected area without disrupting them in order to facilitate removal, in case of future excessive tumor hormone secretion or mutation.

Recently, stem cell-derived cell therapies have been developed, commercialized, and clinically used; these were designed to treat physical defects (in cartilage, Cartistem®; skin, Cupistem®; the cornea, Honoclar®) and to modulate the immune system (amyotrophic lateral sclerosis; Corestem®). Umbilical blood-derived mesenchymal stem cells, autologous adipose-derived mesenchymal stem cells, and autologous bone marrow-derived stem cells were used as cell sources. Hyaluronic acid and collagen have been applied as scaffold material with cells in space-filling cell therapy, and these scaffolds were ideal for physically reconstructing defected areas; however, they were not suitable as hormone-releasing cell carriers because sufficient angiogenesis around the cell is mandatory for secreting the hormone throughout the whole body. Insulin-secreting cells have also been introduced as hormone-releasing cell therapy^16,17^. Cell-protecting polymers, hydrogels, meshes, and devices have been transplanted together to improve the tissue engraftment, but these materials and devices lacked growth factors; as such, various factors including vascular endothelial growth factor, fibroblast growth factor, and hepatocyte growth factor were added to facilitate cellular engraftment^16^. Furthermore, these exogenous materials caused inflammatory reactions; early reactions caused angiogenesis and fibroblast aggregations by numerous cytokines, whereas late reactions could induce the fibrotic capsule surrounding the transplanted tissue, which blocks the hormone release and nutrient supply. Two-staged transplantation was introduced to prevent these late-stage foreign body reactions^18^, but these treatments required second-stage procedures that caused patients discomfort.

Plasma gel has been demonstrated to be safe as an autologous derivative; other researchers have reported on clinical applications of PG as vocal fold injection material and tooth extraction site filling material^11,12,14,19^. Wound healing was also accelerated with PG injection. The amount of gel injected into the body gradually decreased and it was confirmed that it disappeared at 6 months. Histological evaluation confirmed that 60–80 percent of the initial injection dose remained at 3 months after the implantation^12^. Studies have reported minor inflammatory reactions but no severe adverse effects, and late-stage severe foreign body reactions that caused fibrotic encapsulation have not been observed^12,14^. Because the plasma harvesting and manufacturing have less harm to the donor, plasma gels are advantageous for clinical applications.

Authors have reported on the possibility of treating hypoparathyroidism with tonsil-derived stem cells. Among the multiple materials that have been used as cell carriers in these treatment studies, Matrigel® has shown the most promising results in that it provided both an excellent gel microenvironment and sufficient growth factors derived from origin cells. Despite its highly promising *in vivo* experimental results, Matrigel® limits the clinical application of TMSCs because it is derived from tumor cells and cannot be injected into the human body.

Researchers have also examined *in vitro* the functions of plasma in both gel and sol formulations as cell differentiation and culture media^9,10,15^, and authors of previous studies have analyzed the morphological and rheological properties of plasma gels. However, unlike with the present study, for which we fabricated a gel by heating plasma, these previous researchers required additional gelation procedures such as extracting hyaluronic acid, mixing additional chemical agents, and synthesizing hydrogel microbeads^9,10,13,19,20^. These extra processes could incur additional time and costs to the patients.

Reports have been published on animal experiments that used plasma gel as a cell-delivery carrier^20^. Authors of these studies used stem cells and plasma gel as spatial reconstruction material for bony and cartilage defects, and PGs have proven to be proper scaffolds for bone differentiation and subsequent bone formation due to their abundant growth factors and cytokines. However, no studies to date have reported on the effects of plasma gel on endogenous cell differentiation and hormone secretion.

An ideal cell-carrying scaffold should be porous to support oxygen and nutrient delivery from the surrounding tissues; in our study, we observed under SEM that the plasma gel had multiple pores of 10-nm diameter, too small for cell migration but sufficient for oxygen and nutrient supply for cell survival. In terms of scaffold properties, the gel formula was the most advantageous when we injected cells while simultaneously holding them. Our rheological evaluation showed that the plasma gel had modulus as a gel rather than a sol at human body temperature (37 °C). During the experiment, the PG readily passed through the thin 27-gauge needle when we injected it with a syringe, and it remained in the injection site without scattering for two months.

An important function of cell-delivering scaffolds is to prevent cells from dispersing into the blood and prevent damage from phagocytic cells. Scaffolds also have to keep the cells in the implantation site so the cells could do their own-functions. Our results show that the plasma gels performed these functions excellently. When we transplanted the TMSCs with the plasma gel, we could observe yellowish tissue at the transplantation sites with naked eyes. The results of H&E stain could not confirm the existence of the parathyroid gland because we implanted parathyroid hormone releasing ‘cells’ not ‘tissue’. However, immunohistochemistry analysis confirmed that PTH- and CHGA-positive cells had aggregated at the implantation sites as PTH-releasing tissue. Immunofluorescent staining also confirmed the existence of PTH-secreting cells in the transplantation areas. We could not measure PTH in TMSCs alone transplanted groups. We observed increased PTH levels in the groups transplanted with TMSCs and PG for all period of the experimentation. These results demonstrate that PTH-releasing cells could not be engrafted when they were injected without a scaffold. Plasma gels appear to do its function well as scaffolds because they are rich in cytokines, and their physical properties play a role in protecting cells from scattering. Although the individual PTH increases varied among the each experimental animal, this uneven expression could be originated from uneven cellular distribution in the scaffold material. Restoration of the PTH led to recovery of physiological function. Since calcium-included diet was supplied to the animal, hypocalcemia-related death were not observed in all groups. In the TMSC-PG groups, the serum calcium was higher and the phosphorus was lower than the levels in the cTMSC, dTMSC, and PG groups (Table 2). The calcium and phosphorus levels of the PG, dTMSC, and cTMSC groups were not statistically different. These results demonstrated that combining TMSCs with PG effectively improved the pathophysiology of hypoparathyroidism.

It is interesting that even the non-differentiated TMSCs with PG improved PTH restoration in PTX animals. The reason for this result is unclear, but *in vivo* differentiation of TMSCs into parathyroid-releasing cells or stimulation of secondary PTH-releasing organs such as the thymus could be suggested hypotheses^21–23^. Among the two cell types (cTMSC and dTMSC) that were implanted with PG in this study, the intact PTH level restoration rate was 90% for the group transplanted with dTMSC, whereas the group transplanted with cTMSC showed a recovery rate of 40%. These results indicated that TMSCs that were differentiated into parathyroid-releasing cells *in vitro* released PTH more stably. This result corresponds well with results from earlier experimental studies using Matrigel® and spheroid-shaped cells^7,24^. These findings implied that the *in vitro* differentiation of TMSCs into parathyroid-releasing cells may be essential for the stable, *in vivo* function of implanted cells.

It should be noted that future research is needed in this area to address the limitations of this study. First, a long-term study is required to determine the ideal re-injection timing for the hypoparathyroidism treatment. The plasma gel remained in the body less than 6 months, and the iPTH level was evaluated 12 weeks after the single transplantation; the efficacy of this dTMSC-PG injection treatment cannot be fully clarified through this single study. For a clinical advanatage, a long-term study with a repetitive injection study design should be conducted. We also found that the expression level of PTH has a wide range, as iPTH is a hormone present at a very small amount (pg/mL). To verify the results of this study, a large animal study should follow.

We have evaluated the feasibility of using a plasma gel scaffold to treat hypoparathyroidism. The study results indicate that PG is human-applicable, easily injectable, and cell-friendly material. Administration of PTH-secreting tonsil-derived stem cells with plasma gel is highly feasible treatment modality for treating hypoparathyroidism patient.

## Material and Methods

### Isolating and differentiating the TMSCs

TMSC isolation and differentiation into parathyroid hormone secreting cells were conducted as previously described^7,24,25^. Briefly, we harvested tonsil tissue from a single donor (a five-year-old boy) during tonsillectomy. Informed written consent was obtained from the patient

and the study protocol was approved by the Ewha Womans University Medical Cneter institutional review board (ECT 11-53-02). After filtering the chopped and digested tissue, we obtained adherent mononuclear cells by FicollePaque (GE Healthcare, Little Chalfont, UK) density gradient centrifugation. Then, we plated the cells at a density of 1 × 10^8^ cells in a T-150 culture flask in DMEM containing high (4500 mg/L) glucose (Welgene Inc., Korea), 10% FBS (Invitrogen), 100 mg/mL streptomycin, and 100 U/mL penicillin. After 48 h, we removed the non-adherent cells from the medium and replenished the adherent mononuclear cells (the TMSCs) with new culture medium; all TMSCs that we used in this experiment were passage 5. We obtained informed written consent from the patient’s legal guardians, and the EUMC institutional review board approved the study protocol. We differentiated the TMSCs into parathyroid-like cells using the modified Bingham protocol^26^. Briefly, we incubated the cells in DMEM with 10% FBS until they reached 90% confluence and then changed the medium to a differentiation medium that contained activin A (100 ng/mL) and soluble sonic hedgehog (100 ng/mL); we changed this differentiation medium every 3–4 days for 14 days.

### Preparation of plasma gel

We collected venous rat blood from left internal jugular vein in anticoagulant citrate dextrose tubes (BD Diagnostics, Franklin Lakes, NJ, USA) and centrifuged each tube for 15 minutes at 3000 RPM, separating the blood into three layers (red cells, white cells, and plasma, from bottom to top). We aspirated just the plasma layer using a sterile injection bottle attached to a dental syringe and conducted the gelation using a heating machine (ALSA S-1, Genexel-Seine, Seoul, Korea) that heated and cooled the plasma in a sinusoidal pattern to 100 °C for 12 minutes and to 18 °C for 6 minutes.

### *In vitro* characterization of plasma gel

Fabricated gels readily passed through the thin, 27 gauge needle for dental syringe. The fabricated plasma gels passed readily through the thin, 27-gauge dental syringe needle. We examined the gels’ microstructure using a field emission scanning electron microscope (FE-SEM, JSM-6700F, JEOL, Tokyo, Japan), freeze-drying the samples using liquid nitrogen (−196 °C), and mounting them on the FE-SEM stand after coating them with a platinum sputter coater (208HR, Cressington Scientific Instruments, Watford, UK). We investigated the PGs’ moduli using dynamic rheometry (Rheometer RS1, Thermo Haake, Germany) at 37 °C, placing the gels between parallel plates of 25 mm diameter at 0.5 mm gaps. During the dynamic mechanical analysis, we placed the samples inside a chamber with water-soaked cotton to minimize water evaporation. We collected the data under controlled stress (4.0 dyne/cm^2^) at a frequency of 1.0 rad/s. For the histological evaluation, we embedded the fabricated gels in paraffin blocks and stained them with hematoxylin and eosin (H&E).

### *In vivo* animal experiment design

We randomly allocated 60 male Sprague-Dawley rats (Orient Bio, Sungnam, Korea), approximately 8 weeks of age and weighing 260–350 g, into six groups by administered cell type and scaffold. Parathyroidectomy was performed on five groups and sham operation was performed in one group (sham group). In four PTX groups, we administered 1 × 10^6^ TMSCs that we had prepared both without (control cells, cTMSCs) and with differentiation into parathyroid cells (differentiated cells, dTMSCs) and both with and without the PG. In one PTX group, we administrated only PG (plasma gel, PG). The sham group was remained untreated. This design gave us the six experimental groups: sham, PG, cTMSC, dTMSC, cTMSC-PG, and dTMSC-PG.

All animals were acclimated for at least seven days before the experiments, housed under a 12 h light/dark cycle and allowed free access to food and water. We supplied an AIN-93G diet formula (Research Diets, New Brunswick, NJ, USA) that contained 5 g/kg calcium (0.5%) for all the animals during the acclimation and experimental periods^27^. The animal care followed the Guide for the Care and Use of Laboratory Animals by the Institute of Laboratory Animal Resources and the National Institutes of Health and the Animal Experiment Guidelines of Ewha Womans University Medical Research Institute. This study was approved by the Committee for Ethics in Animal Experiments, Ewha Womans University Medical Research Institute.

### Development of hypoparathyroidism rats and administration of the TMSCs

Hypoparathyroidism animal models were developed as previously described^5,27^. Briefly, two hours after intraperitoneal 5-animolevulinic acid (5-ALA) solution injection, the animals were anesthetized by intraperitoneal injection with Zoletil (Virbac Korea, Seoul, Korea) and xylazine chloride (Bayer Korea, Seoul, Korea; 1:1 mix, 0.1 mL/100 g). After vertical skin incision and thyroid gland exposure, we could detect the red fluorescent parathyroid glands under xenon light (390–440 nm) illumination with an ultraviolet filter, and we removed the identified glands. We closed the skin incisions with non-absorbable 4–0 Ethilon® sutures (Johnson & Johnson, New Brunswick, NJ, USA). The same procedure was performed in sham group, except removing procedure of the parathyroid glands.

After we made the vertical skin incisions at each dorsum, the acriotrapezius muscle was exposed, and in the cTMSC and dTMSC groups, we injected the cells into the muscle. For the cTMSC-PG and dTMSC-PG groups, we mixed the 0.5 mL gel and cells in microtubules and administered them by intramuscular injection. The 0.5 mL of PG was administered in PG group in same way. We used a 22-gauge needle for all injections to prevent cell damage, and to identify the implantation sites, we tagged the muscles using the 4–0 Ethilon® sutures.

### Laboratory evaluation

We conducted our laboratory evaluations using animal serum collected by jugular vein puncture 3 days before surgery and day 1, 3, 7, 10, 14, 21, 28, 42, 56, and 84 after implantation. We measured intact PTH, calcium, and phosphorus levels using an enzyme-linked immunosorbent assay (ELISA) (Rat Bioactive Intact PTH ELISA kit, Immutopics, San Clemente, CA, USA) and an automatic chemistry analyzer.

### Histological evaluation

All animals were sacrificed 12 weeks after the surgical procedures. For the histological evaluation of the implanted cells, we removed the muscles that had been marked with the sutures and stored them in neutral buffered formaldehyde embedded in paraffin blocks. After deparaffinization and alcohol rehydration, we conducted H&E staining. Immunohistochemical staining was performed using an automated immunostainer (LEICA BOND-MAX, Leica Biosystems Newcastle Ltd, Newcastle, UK) according to the manufacturer’s protocol. The following primary antibodies were used for immunohistochemical staining: anti-parathyroid hormone (PTH), mouse monoclonal (1:200 dilution), clone 105G7, IgG2a, Novocastra, Newcastle, UK; anti-chromogranin A (CHGA), mouse monoclonal (1:200 dilution), clone LK2H10, IgG1, Novocastra, Newcastle, UK.

For the immunofluorescence analysis, we then incubated the specimens with anti-PTH antibody diluted in bovine serum albumin buffer (1:100, Ab Frontier, Seoul, Korea) and anti- CHGA mouse polyclonal antibody (1:100; Ab Frontier) overnight at room temperature, washed them with 0.1% NP-40 in PBS, incubated them further with Alexa 488-conjugated goat anti-rabbit antibody (Molecular Probes, Inc., Eugene, OR, USA) for 16 h at 37 °C, and counterstained them with DAPI (Pierce, Rockford, IL, USA). We analyzed the preparations with a Leica TCS-SP5 confocal microscope (Leica Microsystems, Wetzlar, Germany).

### Statistical analyses

We performed all statistical analyses using SPSS version 19 (IBM, Chicago, IL, USA), and the results are expressed as means ± standard deviations. We used repeated-measures ANOVA to determine the statistical significance of the weight changes in the groups and the Mann-Whitney U test to determine statistical significance between two groups. We considered a *p* value < 0.05 significant.
